# Quantitative Assessment of Liver Function Using Gadoxetate-Enhanced Magnetic Resonance Imaging: Monitoring Transporter-Mediated Processes in Healthy Volunteers

**DOI:** 10.1097/RLI.0000000000000316

**Published:** 2016-12-19

**Authors:** Leonidas Georgiou, Jeffrey Penny, Glynis Nicholls, Neil Woodhouse, François-Xavier Blé, Penny L. Hubbard Cristinacce, Josephine H. Naish

**Affiliations:** From the *Centre for Imaging Sciences, and †Manchester Pharmacy School, University of Manchester, Manchester; ‡AstraZeneca Research and Development–DMPK Innovative Medicines, and §AstraZeneca Personalised Healthcare and Biomarkers iMED, Melbourn, Royston, United Kingdom.

**Keywords:** contrast agent, DCE-MRI, gadoxetate, Primovist, tracer kinetic model, liver, clinical, uptake, efflux, pharmacokinetics

## Abstract

**Objective:**

The objective of this study was to use noninvasive dynamic contrast-enhanced magnetic resonance imaging (MRI) techniques to study, in vivo, the distribution and elimination of the hepatobiliary contrast agent gadoxetate in the human body and characterize the transport mechanisms involved in its uptake into hepatocytes and subsequent efflux into the bile using a novel tracer kinetic model in a group of healthy volunteers.

**Materials and Methods:**

Ten healthy volunteers (age range, 18–29 years), with no history of renal or hepatic impairment, were recruited via advertisement. Participants attended 2 MRI visits (at least a week apart) with gadoxetate as the contrast agent. Dynamic contrast-enhanced MRI data were acquired for approximately 50 minutes with a 3-dimensional gradient-echo sequence in the axial plane, at a temporal resolution of 6.2 seconds. Data from regions of interest drawn in the liver were analyzed using the proposed 2-compartment uptake and efflux model to provide estimates for the uptake rate of gadoxetate in hepatocytes and its efflux rate into the bile. Reproducibility statistics for the 2 visits were obtained to examine the robustness of the technique and its dependence in acquisition time.

**Results:**

Eight participants attended the study twice and were included into the analysis. The resulting images provided the ability to simultaneously monitor the distribution of gadoxetate in multiple organs including the liver, spleen, and kidneys as well as its elimination through the common bile duct, accumulation in the gallbladder, and excretion in the duodenum. The mean uptake (*k*_i_) and efflux (*k*_ef_) rates in hepatocytes, for the 2 visits using the 50-minute acquisition, were 0.22 ± 0.05 and 0.017 ± 0.006/min, respectively. The hepatic extraction fraction was estimated to be 0.19 ± 0.04/min. The variability between the 2 visits within the group level (95% confidence interval; *k*_i_: ±0.02/min, *k*_ef_: ±0.004/min) was lower compared with the individual variability (repeatability; *k*_i_: ±0.06/min, *k*_ef_: ±0.012/min). Data truncation demonstrated that the uptake rate estimates retained their precision as well as their group and individual reproducibility down to approximately 10 minutes of acquisition. Efflux rate estimates were underestimated (compared with the 50-minute acquisition) as the duration of the acquisition decreased, although these effects were more pronounced for acquisition times shorter than approximately 30 minutes.

**Conclusions:**

This is the first study that reports estimates for the hepatic uptake and efflux transport process of gadoxetate in healthy volunteers in vivo. The results highlight that dynamic contrast-enhanced MRI with gadoxetate can provide novel quantitative insights into liver function and may therefore prove useful in studies that aim to monitor liver pathology, as well as being an alternative approach for studying hepatic drug-drug interactions.

Magnetic resonance imaging (MRI) is a nonionizing imaging technique that frequently uses paramagnetic contrast agents to evaluate suspected lesions or monitor treatment response.^1,2^ For quantitative analysis, high temporal resolution dynamic images are required to capture the rapid temporal signal changes of the passage of a bolus of contrast agent through the arteries. In addition, signal from the tissue of interest is simultaneously sampled at successive time points. The temporal characteristics of these signal time curves reveal information regarding the absorption, distribution, and elimination of the contrast agent in the tissue of interest and therefore offer the potential to investigate and monitor these processes in vivo.

While some groups of contrast agents remain extracellular after their passive diffusion across the endothelial barrier of capillary walls, others may be taken up or released from specific cell types through transporter-mediated processes. Gadoxetate is a hepatobiliary contrast agent that is known to be a substrate of hepatic transporters and therefore offers the opportunity to examine these transport mechanisms in vivo. Various studies have demonstrated the interaction of gadoxetate with transporter proteins in the liver. Leonhardt et al,^3^ using HEK293 cells that overexpress uptake transporter proteins, showed that gadoxetate is a low-affinity, high-capacity substrate for both the human liver–specific organic anion transporter polypeptide (OATP)1B1 and 1B3, and a high-affinity, low-capacity substrate for the Na^+^-taurocholate cotransporting polypeptide (NTCP), which are expressed at the hepatocyte sinusoidal (basolateral) membrane. In another study, Jia et al^4^ showed that gadoxetate is a low-affinity substrate for the uptake transporter OATP1A2, which is suggested to be expressed in the intestine, and using inside-out vesicles, demonstrated that gadoxetate is a substrate of the human multidrug resistance–associated protein (MRP)2 efflux transporter, which is expressed on the hepatocyte canalicular (apical) membrane. MRP2 was thought to play a role in the hepatic elimination of gadoxetate because earlier preclinical in vivo studies demonstrated that mutant Wistar rats with rodent MRP2 deficiency exhibited a prolonged liver enhancement of gadoxetate signal and reduced biliary gadoxetate elimination, compared with wild-type rats.^5–7^ Another finding was that rodent MRP3 expression was significantly upregulated in the liver of rodent MRP2 deficient rats, compared with the control group. Consequently, while the latter exhibited minimal efflux of gadoxetate out of the hepatocyte across the sinusoidal membrane, enhanced efflux across the sinusoidal membrane was observed in rodent MRP2-deficient rats. Similar findings have also been reported in other studies.^8,9^

During the last decade, gadoxetate-enhanced MRI has been applied clinically for the detection and characterization of focal hepatic lesions.^10,11^ Following an intravenous bolus, 3 distinct phases are observed. In the first 2 phase (arterial and portal venous phase), which occur during the first few minutes, the image contrast and the respective signal time course is similar to that of extracellular agents.^12^ Subsequently, due to the accumulation of gadoxetate in liver cells and its slow excretion into the bile, approximately 20 minutes after contrast administration, a third hepatobiliary phase is reached. Clinical protocols typically utilize carefully timed acquisitions of T1- and T2-weighted images during each of these 3 distinct phases to characterize a variety of liver lesions (eg, simple cyst, hemangiomas, adenoma, focal nodular hyperplasia, metastases from colorectal cancer).^11,13–17^ A series of studies have also monitored the time course of contrast agent accumulation and appearance in the biliary system and gallbladder (eg, hepatobiliary transit times) as a means to characterize the biliary ductal system.^18–20^

Liver function, as determined via quantitative dynamic contrast-enhanced (DCE) MRI with gadoxetate, has also been used in both humans and animals. Nilsson et al^21,22^ fitted a monoexponential decay to the deconvolved liver response function to estimate a hepatic extraction fraction in healthy human volunteers of 0.21 ± 0.05. Ulloa et al^23^ assessed the potential to detect liver cholestasis (bile formation impairment) in rats by modeling signal decay as a single exponential. Sourbron et al introduced the dual-input 2-compartmental model to describe the uptake of gadoxetate in liver tissue over a period of 5 minutes. This model incorporates the accumulation of contrast agent in the hepatocytes at a linear uptake rate but does not attempt to model efflux, assuming that it is negligible over this short duration.^24,25^ The model was implemented using data from patients with hepatic metastases, and data from regions of tissue with normal appearance and lesions were compared. The results demonstrated that including the uptake rate parameter for the hepatocytes provided a better fit of the time series compared with the 1-compartmental model; however, the variability of the uptake rate estimates highlighted the need for more data to establish the reproducibility of the techniques.

More recently, Ulloa et al^26^ presented a model that incorporates both linear uptake and nonlinear biliary efflux to fit DCE data obtained in rats for an acquisition time of 60 minutes. Both the uptake and efflux processes are governed by nonlinear Michaelis-Menten kinetics, but an attempt to incorporate these in a complex model might be problematic. The maximum number of identifiable parameters in a 2-compartment tracer kinetic model is four,^27^ and hence the need to use 2 parameters to characterize each nonlinear transport mechanism (ie, *k*_m_, *V*_max_) limits any further parameters, making the model incomplete. Furthermore, the use of nonlinear kinetics for either uptake and/or efflux prohibits the use of an analytical solution because although an explicit solution to the Michaelis-Menten equation exists, the differential equations that describe the model have to be solved numerically.^28^ These limitations become more problematic when the acquired data are suboptimal. For example, the low temporal resolution used in Ulloa et al (30 seconds) could further reduce the accuracy of the parameter estimates. The increased complexity of nonlinear kinetics and the spatiotemporal limitations of the acquired data can introduce large uncertainties in the parameter estimates.^29^

During preclinical studies using gadoxetate, Schuhmann-Giampieri et al showed that biliary excretion rate is saturated at doses higher than 0.6 mmol/kg. The clinically accepted dose for gadoxetate is 24-fold lower (0.025 mmol/kg), which is well below the saturation level.^30^ This, in parallel with the technical limitations stated above, suggests that a simple linear approximation for the efflux transport mechanism might be a more suitable approach. In this article, we present a dual-input 2-compartmental model that characterizes both the uptake and efflux of gadoxetate from the liver using a linear uptake and elimination approximation and assesses the reproducibility of the technique for a group of healthy volunteers. We also investigate the applicability of the protocol in a clinical environment by examining the impact of the duration of the acquisition on the precision of the uptake and efflux parameters. Finally, we discuss the potential use of the protocol to simultaneously monitor the distribution of gadoxetate in multiple major organs for a more thorough characterization of the elimination pathway, and also highlight the opportunity for a more detailed assessment of the biliary ductal system.

## MATERIALS AND METHODS

### Tracer Kinetic Model

**FIGURE 1:**
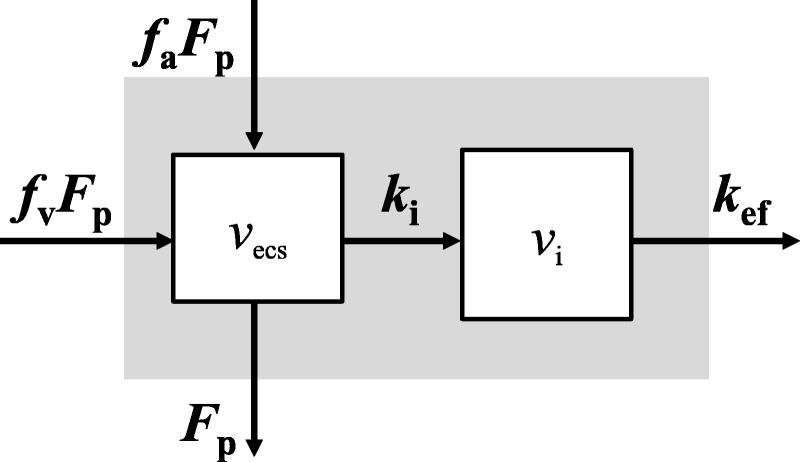
Dual input 2-compartmental uptake and efflux model.

The model presented in this article (Fig. 1) is a dual-input 2-compartmental uptake and efflux model, an extension of the uptake model of Sourbron et al,^24^ that describes the pharmacokinetic properties of gadoxetate in the liver for a prolonged acquisition. The model assumes that the liver parenchyma comprises (1) the extracellular space, which consists of the vascular (ie, plasma) and interstitial space, *v*_p_ and *v*_e_, respectively, and (2) the intracellular space *v*_i_ (ie, hepatocytes). The contrast agent reaches the liver parenchyma through a dual pathway, and the contribution of each route to the total input was assumed to be the weighted sum of the arterial supply fraction, *f*_a_ (ie, hepatic artery), and venous supply fraction, *f*_v_ (hepatic portal vein). The exchange of contrast agent between the plasma and interstitial space was assumed to be rapid (ie, endothelial permeability→∞) and to immediately reach equilibrium.^31,32^ The extracellular space was therefore assumed to behave as a single compartment, *v*_ecs_. Gadoxetate was assumed to enter the hepatocytes via a unidirectional linear transport mechanism, *k*_i_, with excretion back to the sinusoids being negligible.^4,8,9^ It is known that the contrast agent is eliminated from the circulation by outflow, through the kidneys (~50%) and by active transport into the bile (~50%). A unidirectional linear efflux of contrast agent from the intracellular space to the bile canaliculi with a transfer rate *k*_ef_ was assumed. In addition, because the volume of the bile canaliculi is extremely small compared with the overall extracellular and intracellular volumes, its contribution to the measured tissue concentration was assumed to be negligible; bile is also continuously carried away from the liver through the hepatic bile ducts toward the common bile duct.^33,34^ The model is defined in Equation 1 (see Supplementary Text Document, Supplemental Digital Content 1, http://links.lww.com/RLI/A297, for the derivation):

**Formula:**
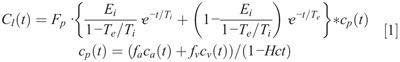


The dual-input 2-compartmental uptake and efflux model described in Equation 1 yields the following parameters: plasma flow rate, *F*_p_; extracellular mean transit time, *T*_e_ (*v*_ecs_/[*F*_p_ + *k*_i_]); the intracellular mean transit time, *T*_i_ (*v*_i_/*k*_ef_); the hepatic uptake fraction, *E*_i_ (*k*_i_/[*F*_p_ + *k*_i_]); and the arterial fraction, *f*_a_. It should be noted that the hepatic portal venous fraction is estimated as 1 − *f*_a_. To estimate the efflux rate, *k*_ef_, the intracellular volume is required. Since the intracellular space of the liver consists mostly of hepatocytes, with all other cell types occupying a negligible volume,^33,35^ the intracellular volume was therefore estimated as 1 − *v*_ecs_. Furthermore, the hematocrit level (*Hct*) was assumed to be 0.45 for all participants.^36^

### Participants

Ten healthy volunteers were recruited via advertisements. All participants had no history of renal or hepatic impairment. The study was approved by the university's ethics committee, and written informed consent to undergo DCE-MRI of the liver with contrast injection was obtained from all participants before examination. All volunteers were screened for MR exclusion criteria, such as pacemakers and metal implants, and for serious illness or surgery that might affect the study results. To study the reproducibility, participants were formally asked to attend 2 visits at least 1 week apart. This was decided based on the mean terminal elimination half-life of gadoxetate observed in healthy subjects, which is 1 hour; therefore complete elimination of the contrast agent was expected.^37^

### Data Acquisition

The volunteers were imaged on a 1.5 T MRI scanner (Philips Achieva) using the inbuilt body coil. Precontrast sequences included 4 axial 3-dimensional T1-weighted images at variable flip angles (2, 10, 20, and 30 degrees) for T1 quantification and had the same parameter settings as the dynamic MR sequence, except for the flip angle. Dynamic contrast-enhanced MRI data were acquired for 50 minutes at a temporal resolution of 6.2 seconds and a flip angle of 20 degrees, with a 3-dimensional RF-spoiled gradient-echo sequence (T1 fast field echo). The imaging parameters for both were as follows: 48 axial slices; voxel size, 2.1 × 2.1 × 4 mm; reconstruction matrix, 176 × 176; 3 milliseconds repetition time; 0.68 millisecond echo time; 88 phase encoding steps; 62% sampling; partial Fourier; and Fourier interpolation. Two minutes after the start of the DCE acquisition, a bolus of gadoxetate (Primovist; Bayer, Leverkusen, Germany) at a clinically relevant dose of 0.025 mmol/kg (ie, 0.1 mL/kg) was administered at 2 mL/s and flushed with 20 mL of saline at the same rate.

### Image Postprocessing

Data were postprocessed using Matlab (R2014a; MathWorks, Natick, MA) and ImageJ. No motion correction was performed. A liver tissue region of interest (ROIs) was defined over several slices within the liver, and a dynamic time series was extracted. An arterial input function (AIF) was generated using a ROI manually defined within the lumen of the abdominal aorta. To minimize inflow effects, the ROIs were outlined on inferior slices that exhibited the highest peak on the corresponding relative signal enhancement curves (*S*(*t*)/*S*_*0*_ − 1).^38^ In addition, only a small number of pixels (eg, 2–4) were selected within each slice, in locations well within the aorta to reduce any partial volume effects. The venous input function (VIF) was extracted from an ROI within the lumen of a branch of the hepatic portal vein. Baseline T1 relaxation time estimates for the liver tissue, the AIF, and the VIF were obtained by fitting the steady-state equation of FLASH sequence to the signal extracted from the variable flip angle images using the respective ROIs. These baseline T1 values were subsequently used to convert the signal intensity time series from the dynamic data to concentration of the contrast agent in the respective tissues, by assuming a T1 relaxivity of gadoxetate of 6.9 mM^−1^s^−1^ at 1.5 T.^39^ The process of converting MR signal into concentration has been described previously.^23^

### Data Analysis

The tracer kinetic model was fitted up to the 50th minute of the acquisition using a least squares fitting algorithm from the Matlab optimization toolbox (Matlab 2014Rb). To assess the reproducibility of the parameter estimates for each participant, the difference between the parameters in each visit was calculated. The method of analysis is described in previous studies.^40,41^ To investigate the dependence of the parameter estimates on the acquisition time, the data were truncated, yielding 46 data sets for each volunteer with acquisition times from 5 to 50 minutes at increments of 1 minute, and the model was fitted to each of the truncated data sets.

The delay between the contrast bolus arrival in the hepatic portal vein and the liver tissue was assumed to be negligible compared with the temporal resolution of the data acquisition.^24^ In contrast, the delay between the contrast bolus arrival in the artery and the liver tissue was determined by fitting the data for the first 5 minutes of the dynamic acquisition with different delay values and choosing the delay with the best fit (minimum sum of squared errors). A short acquisition time of 5 minutes was used to estimate the delay value to ensure that the sum of squared errors is sensitive to the first pass peak and not biased from the prolonged acquisition time. Furthermore, because variable acquisition times were fitted, it was important to ensure that the arterial delay used was fixed based on the same criteria for all series, so that any changes on the parameter estimates were not affected by the choice of arterial delay.

To ensure that the solution lies within physiological values, extended constraints were applied that allowed parameters to vary within that range (0 < *F*_p_ < 5 mL/min per mL, 0 < *f*_a_ < 0.6, 0 < *k*_i_ < 5/min, 0 < *k*_ef_ < 5/min, 0 < *v*_e_ < 0.6 mL/mL). Parameter differences were tested for normality using Shapiro-Wilk test and Kendall τ for dependence of absolute value of the difference, *d*, against the mean value for the 2 visits.

## RESULTS

From the 10 volunteers initially recruited, the final study cohort included 5 men (age range, 18–29 years; mean age, 25 years) and 3 women (age range, 18–22 years; mean age, 20 years). These participants were scanned twice, with the period between each visit being 1 to 4 weeks. For the 2 remaining volunteers, 1 participant interrupted the study during the scan because of claustrophobia and declined a second visit. Another participant attended the study once and did not respond to the request for a second visit. They were therefore not included in the analysis.

The volume slab acquired in this study (Supplementary Figure S2-1, Supplemental Digital Content 2, http://links.lww.com/RLI/A298) demonstrates the organs that could be simultaneously monitored during the distribution of the contrast agent in the body. For a more descriptive report and discussion on the organs and anatomical regions identified during the MRI scan, as well as examples of the respective relative signal intensity (RSI) time series refer to the Supplemental Digital Content 2, http://links.lww.com/RLI/A298.

### Tracer Kinetic Model Fitting and Reproducibility

Baseline T1 relaxation times in the liver, aorta, and portal vein were 595 ± 25, 1163 ± 82, 1226 ± 69 milliseconds, respectively. Because of breathing motion-induced artifacts, it was difficult to obtain a VIF for all participants, and therefore population averages for both AIF and VIF were estimated from 16 and 12 individual input function (both visits), respectively (Supplementary Figure S2-3, Supplemental Digital Content 2, http://links.lww.com/RLI/A298).

**FIGURE 2:**
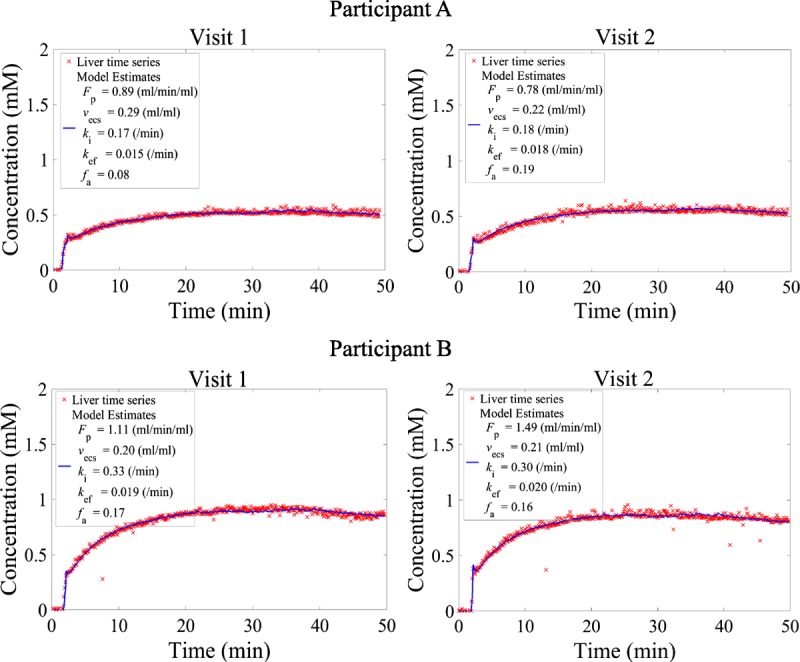
DCE-MRI liver time series fitted to the dual-input 2-compartmental uptake and efflux model shown in Equation 1 for visits 1 and 2 of participants A and B. The red crosses correspond to the acquired data and the continuous blue line to the fitted model.

Examples of model fits to the concentration time series of participants for both visits are shown in Figure 2. The mean value of total plasma flow (*F*_p_) for all participants on both visits was 1.00 ± 0.27 mL/min per mL, the mean liver extracellular space fraction was 0.20 ± 0.05 mL/mL, and the mean arterial flow fraction (*f*_a_) was 0.17 ± 0.12. The mean uptake rate into and efflux rate out of hepatocytes were 0.22 ± 0.05 and 0.017 ± 0.006 min^−1^, respectively.

All parameter differences followed a normal distribution (*P* > 0.05, Shapiro-Wilk test). In addition, Kendall τ for dependence of absolute values of the difference, *d*, against the mean value for the 2 visits did not show any significant correlation, and thus all reproducibility statistics were performed based on the absolute values of each estimate.

**FIGURE 3:**
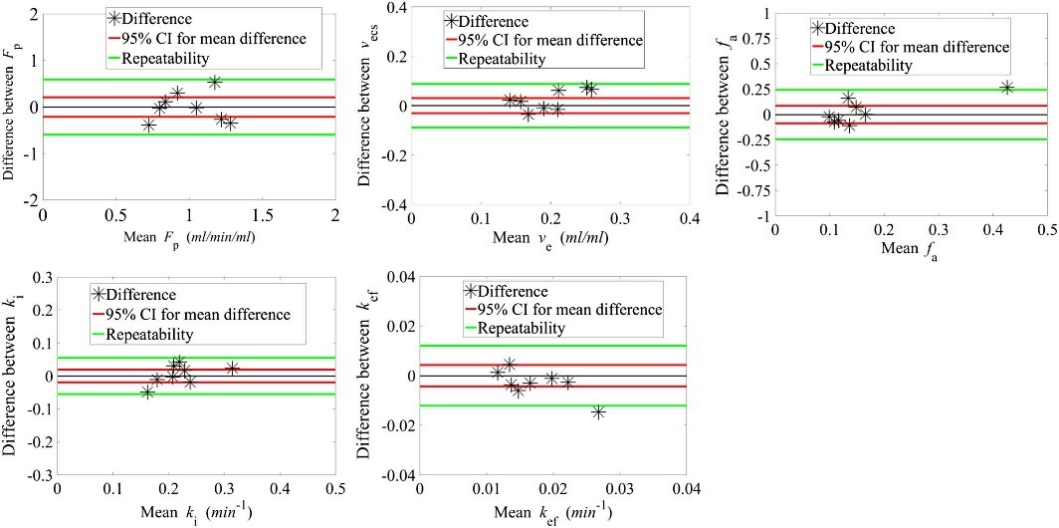
Difference between visit 1 and 2 parameter estimates plotted against the mean value for a 50-minute acquisition liver time series. Red lines correspond to the 95% confidence interval (CI) for a group of 8. Green lines show the repeatability of each parameter for an individual change.

Figure 3 shows Bland-Altman plots of the difference in fit parameters between the 2 scans against the mean values from the 2 scans for each participant. The 95% confidence intervals (CIs) for the difference are also shown with a range of ±0.21 mL/min per mL for *F*_p_, ±0.03 mL/mL for *v*_ecs_, ±0.02 and ±0.004 min^−1^ for *k*_i_ and *k*_ef_, respectively, and ±0.09 for *f*_a_. These measurements indicate the variability for each parameter estimate within a group of 8 healthy volunteers.

**FIGURE 4:**
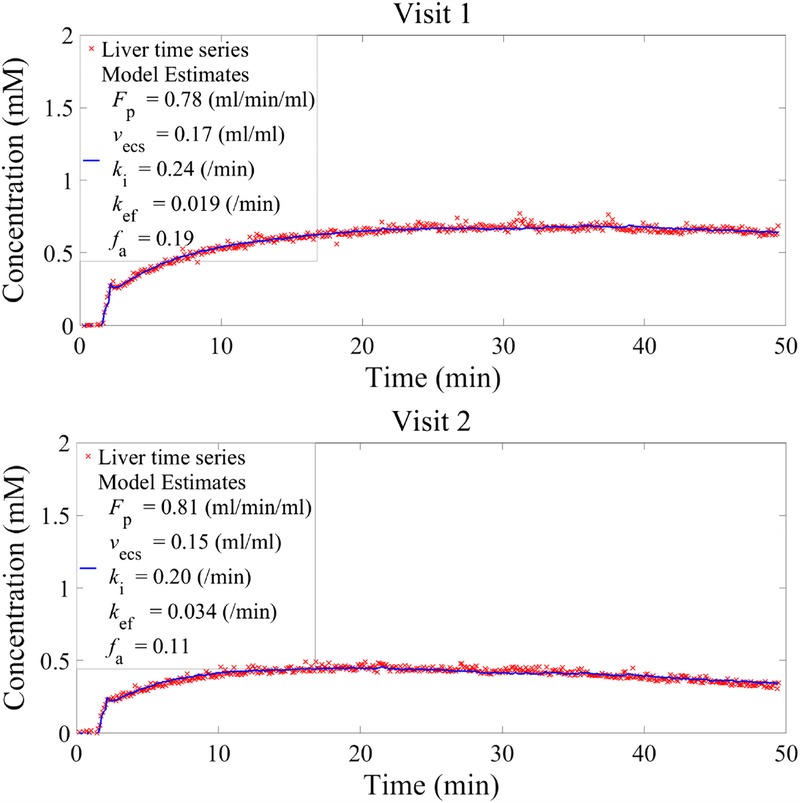
Variability of concentration time series and the efflux parameter between visit 1 and 2 for participant C. The change in *k*_ef_ exceeds the repeatability limits for a single individual.

The mean value for each parameter, the corresponding standard deviation, and the mean difference between visits for all participants are also shown in Table 1. Repeatability values illustrate the extent of fluctuation from the initial estimate that would indicate a significant change for an individual. For example, a change in the efflux rate of more than 0.012 min^−1^ would be classified as significant. The *k*_ef_ plot in Figure 3 illustrates how the difference calculated for 1 participant (participant C) lies beyond the reproducibility limits. The variation was also visible on the liver concentration time series of this participant (Fig. 4), where in visit 2 the corresponding time series exhibited a faster washout compared to visit 1. This was reflected in the *k*_ef_ estimates (0.019 and 0.034 min^−1^ for visit 1 and visit 2, respectively), with the mean difference for all participants being as low as −0.003 min^−1^. Nevertheless, the group reproducibility for the efflux parameter is higher than for the individual reproducibility.

**TABLE 1:**
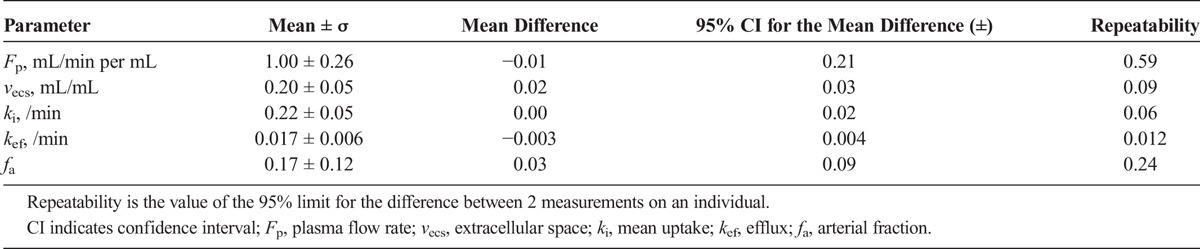
Estimated Tracer Kinetic Parameters and Their Reproducibility for a 50-Minute Acquisition Time

The least reproducible parameter on both a group and an individual level was *f*_a_, the arterial supply fraction (±0.09 and ± 0.24, respectively). Conversely, the most reproducible parameters were the uptake rate into hepatocytes and extracellular space fraction, with the repeatability being sensitive to changes of the order of 0.06 min^−1^ and 0.09 mL/mL for *k*_i_ and *v*_ecs_, respectively. The reproducibility on a group level was even higher, with 95% CI of 0.02 min^−1^ and 0.03 mL/mL, respectively.

### Effect of Data Truncation on Parameter Estimates

**FIGURE 5:**
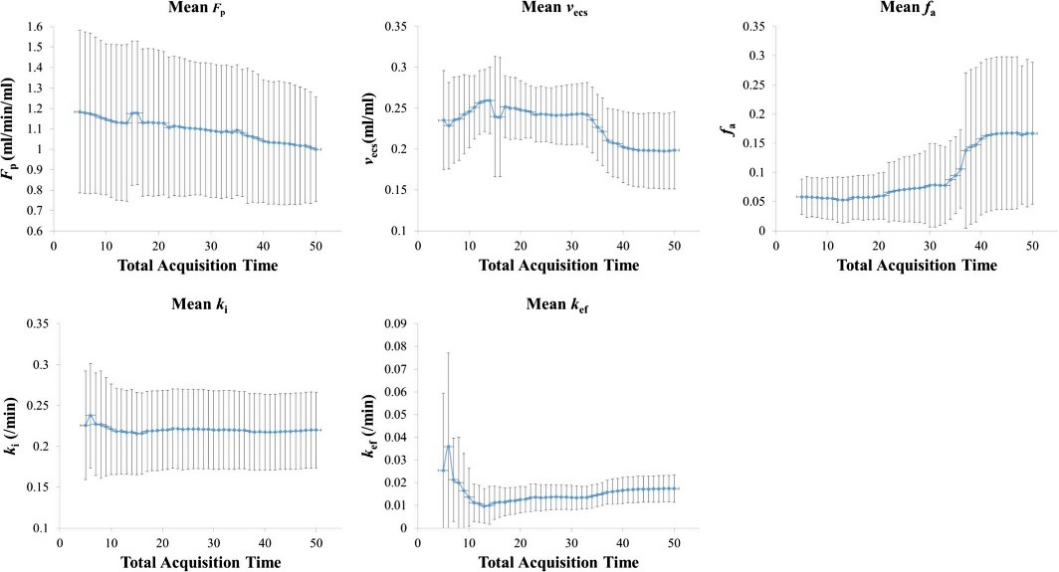
Plots of the means and standard deviations (σ) of the tracer kinetic model estimates (*F*_p_, *v*_ecs_, *k*_i_, *k*_ef_, *f*_a_) against acquisition times.

**FIGURE 6:**
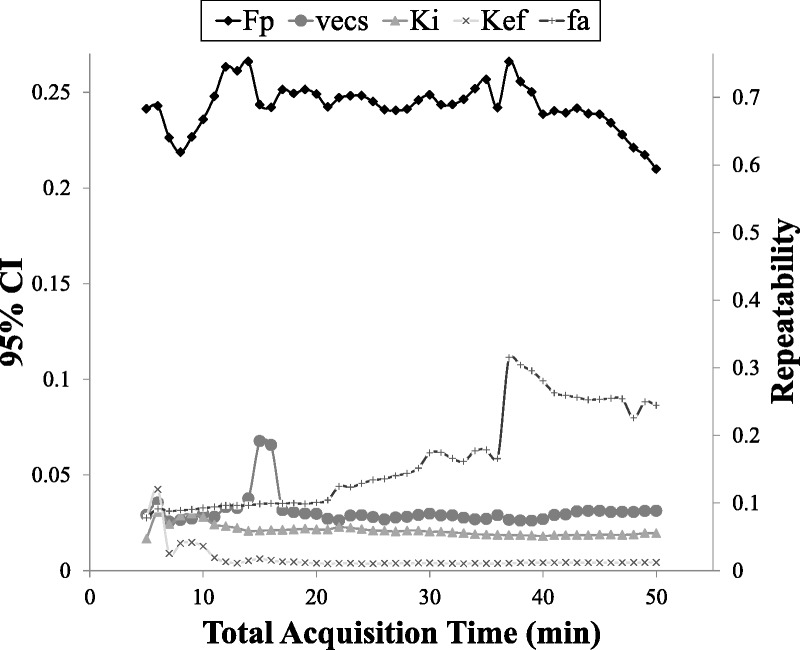
Plots of the 95% CI and repeatability of the parameter estimates against acquisition times.

Mean values of the parameter estimates (*F*_p_, *v*_ecs_, *k*_i_, *k*_ef_, *f*_a_) from all volunteers were plotted as a function of acquisition time as shown in Figure 5, along with the corresponding standard deviation. The reproducibility statistics (95% CI and repeatability) for each parameter are also shown in Figure 6.

For the components of the model that reflect rapid processes, that is, *F*_p_ and *k*_i_, the mean estimates did not suffer large variations over time; however, slightly higher variations in the mean estimates were observed at shorter acquisition times (eg, 5 minutes) compared with longer acquisition times (eg, 50 minutes), as observed from Figure 5. The mean *k*_i_ estimates remained relatively constant even at the shortest acquisition time (5 minutes). The mean *F*_p_ estimates increased slightly while moving to shorter acquisition times. The mean *v*_ecs_ estimates had a slightly higher variability compared with *F*_p_ and *k*_i_ as the acquisition time decreased. However, the 95% CI and repeatability remained approximately constant even at lower acquisition times, apart from a sudden fluctuation at an acquisition time around 15 minutes.

In contrast, the slow component of efflux process, *k*_ef_, demonstrated a higher variability at shorter acquisition times compared with longer times. The corresponding 95% CI and repeatability remained relatively constant between acquisition times of approximately 10 and 50 minutes; however, higher variability in parameter estimates is observed below 10 minutes. In terms of the actual mean estimates, these were increasingly underestimated (with respect to the 50 minutes acquisition time point) down to 13 minutes of acquisition (0.009 ± 0.0075 min^−1^) and then overestimated (at the sixth minute of acquisition) where *k*_ef_ was 0.036 ± 0.041 min^−1^. The mean arterial fraction, and associated variability, was seen to decrease with decreasing acquisition time.

## DISCUSSION

### Tracer Kinetic Model and Reproducibility Study

Liver T1 estimates were in good agreement with those reported in the literature (586 ± 39 milliseconds^42^). Blood T1 estimates were lower compared to literature values (1400 milliseconds^43,44^), likely due to uncorrected inflow and partial volume effects, respectively. The tracer kinetic modeling results obtained by fitting the dual-input 2-compartmental uptake and efflux model to the 50-minute acquisition provided a good description of the transport of gadoxetate into, and out of, hepatocytes. The estimated rates reflect the overall transport rate of a combination of transporters expressed on both the sinusoidal (eg, the uptake transporters OATP1B1, OATP1B3, and NTCP) and the apical (eg, the MRP2 efflux transporter) membranes.

The results indicate that the model generates a robust and reproducible cellular uptake rate, *k*_i_. In addition, total plasma flow (*F*_p_), efflux rate (*k*_ef_), extracellular space (*v*_ecs_), and arterial flow fraction (*f*_a_) could be estimated. The 95% CI and repeatability limits are good indicators of the variability of each parameter in a group and at an individual level, with *v*_ecs_ and *k*_i_ showing the least variability. The efflux rate estimates were less reproducible at the individual level, compared with the uptake rate (*k*_i_). This may be attributed to the 50-minute acquisition time not being sufficient to provide a robust estimate of the efflux process. The concentration time series patterns for visit 1 and visit 2 were found to be similar for most volunteers. Interestingly, within the group of healthy volunteers, one of the participants exhibited a different liver concentration time series pattern in visit 2 compared with visit 1, both qualitatively (Fig. 4 participant C; peak concentration was reached at an earlier time point, and subsequent wash-out appears more rapid in visit 2) and quantitatively. The reason for the observed variability is unknown. Although all participants attended the study within 2 weeks, the gap between the 2 visits for this particular participant was 4 weeks. The observed difference might be attributed to physiological changes within that period. Although the corresponding repeatability values are significantly improved by excluding participant C (0.005 min^−1^), the 95% CI demonstrate that the reproducibility within the group as a whole remains high even with this participant included.

To our knowledge, this is the first attempt to quantify uptake rate into and efflux rate out of hepatocytes using gadoxetate in healthy volunteers, and hence no standard reference values were available for comparison. As mentioned earlier, reference uptake rate estimates have been obtained from a study in patients with hepatic metastases using a dual input 2-compartmental uptake model with DCE-MRI.^24^ The RSI time series generated from normal-appearing liver yielded a mean uptake approximately 7-fold lower than the mean uptake rate estimated in this study. Three possible reasons may have influenced this; (1) the Sourbron model was fitted on the RSI data only and hence does not account for the nonlinear relation between signal intensity and concentration at high concentrations, (2) the data were assumed to reflect normal (nondiseased) liver from compromised patients, and (3) these patients were also undergoing treatment. Particularly for the latter, drug-drug interactions (DDIs) at the level of OATP transporters may alter the pharmacokinetic profile of gadoxetate in the liver parenchyma and, as shown in preclinical studies, can cause a reduction in the uptake rate estimates.^26^

Estimates for *F*_p_, *v*_ecs_, and *f*_a_ were consistent with the values reported in the literature.^45–47^ Furthermore, using the individual participant estimates of *k*i and *F*_p_, the hepatic extraction fraction was estimated to be 0.19 ± 0.04 min^−1^, which is in very good agreement with that reported by Nilson et al (0.21 ± 0.05 min^−1^) who used the deconvolved liver response function and fitted a monoexponential to the resulting hepatic extraction curve.^21,22^

### Effect of Data Truncation on Parameter Estimates

The tracer kinetic model was initially fitted to the 50-minute acquisition time to establish the reproducibility of the parameter estimates, particularly for the uptake and efflux rates of gadoxetate. However, although in this study, ethics approval was obtained to perform a prolonged acquisition time (to acquire as much information from the signal time series as possible), a shorter acquisition time would be preferable in clinical practice in terms of both cost and patient tolerability.

As expected, the model parameters that reflect the physiological processes that occur at the early stages of dynamic acquisition following bolus administration, that is, *F*_p_ and *k*_i_, exhibited the least variation as the acquisition time (*T*_acq_) was decreased. The plasma flow estimates demonstrated a slow increase in the mean value with an increase in standard deviation as *T*_acq_ decreased. However, the uptake rate remained relatively constant for different acquisition times.

The extracellular space fraction estimates were slightly overestimated with respect to the 50-minute acquisition time and showed some instability and higher uncertainty at acquisition times less than 15 minutes. This is expected because the accuracy of *v*_ecs_ estimates in general is governed by the length of acquisition (related to the area under the impulse response function), which is uncertain if insufficient time is allowed for function decay.^31^ Nevertheless, the *T*_e_ estimate (the combination of *v*_ecs_, *F*_p_, and *k*_i_) was robust, even for an acquisition time of 5 minutes, because the extracellular transit time was of the order of tens of seconds.

The acquisition time became more important in the case of efflux rate estimates. The uncertainty in *k*_ef_ was expected to increase at lower acquisition time because the intracellular residence time is of the order of tens of minutes (a rough estimation using the 50-minute acquisition suggests that this is approximately 45 ± 15 minutes). To precisely estimate *k*_ef_, the acquisition time needs to be high enough to capture this residence time. Furthermore, interindividual variability plays a major role in the precision of efflux rate estimates. Nevertheless, the mean parameter estimate for efflux and the corresponding standard deviation beyond the 13th minute was stable and slightly increased toward higher *T*_acq_. The respective 95% CI and repeatability limits exhibited the same characteristics. This suggests that independent of the accuracy of the efflux rate, the precision was stable. The estimated reproducibility at the group level should be high enough to be able to sensitively detect changes to this parameter for a similar sized group of patients with biliary impairment.

As the number of healthy volunteers recruited was small, a larger study cohort would be necessary to thoroughly test the model and establish the reproducibility of the techniques in a large population of healthy individuals. In this work, no motion correction was performed. Although this did not compromise the high CNR of liver concentration time series, it led to difficulties in obtaining a signal from the hepatic portal vein, the major blood supply to the liver. This had an impact on the choice of individual or population average input functions, which may prove important in some studies. For example, the use of an individual input function would be essential in DDI studies because the inhibitory effect of the coadministered drug might vary within the population of the study.

### Implications

The imaging volume acquired in this study allowed simultaneous monitoring of the common bile duct (CBD) and the gallbladder. These may provide additional information regarding the physiological processes that occur at the apical membrane of hepatocytes. Any signal observed from these anatomical regions would be due to gadoxetate efflux from hepatocytes. A more detailed study into the physiology of the CBD and gallbladder could therefore improve the estimated values for efflux, even at shorter acquisition times. These signals may also be utilized in the study of biliary diseases.^48,49^

The reproducibility of the technique provides good evidence to support future studies that could have significant implications in clinical practice. Ulloa et al,^26^ for example, demonstrated how DCE-MRI techniques can quantify DDIs at the transporter-protein level in rat models. This highlights the potential use of gadoxetate to provide valuable information regarding hepatic DDIs in both animals and humans. A variety of drugs, such as statins, immunosuppressant (eg, cyclosporin), rifampicin, and its variants, and even food substances, such as apples, oranges, and grapefruit juice, could have a substantial impact on the pharmacokinetics of gadoxetate because they may inhibit its transport in hepatocytes.^50^

Furthermore, it has been observed that some genetic polymorphisms of the uptake transporter OATP1B1 (*SLCO1B1*) can alter the uptake rate of gadoxetate in vitro.^51^ These genetic variants were also correlated with alterations in liver enhancement of MRI scans. Carriers of specific SLCO1B1*5 variants have shown a significantly reduced liver enhancement (30%–40%) of gadoxetate, indicating a loss of function compared with the wild-type transporter.^52^ Moreover, reduced expression of OATP transporters has been reported to be associated with hepatocellular carcinomas, adenomas, and hepatitis C virus–related cirrhosis.^53–55^ Gadoxetate may therefore deliver quantitative insights into liver pathology and provide a more objective assessment of liver function and thus play a key role in clinical decision making, through preoperative and postoperative evaluation of the liver.^56–59^

## CONCLUSIONS

In conclusion, this study reports the first tracer kinetic model that describes liver function in terms of both the uptake rate of gadoxetate into hepatocytes and subsequent efflux into the bile, in healthy volunteers. In addition, we explored the reproducibility of the techniques used by performing the measurements twice on the same subjects. Using the proposed imaging protocol, we have also been able to extract continuous signal time series that demonstrate the elimination of gadoxetate in the CBD, gallbladder, and duodenum, at a high temporal resolution, which to our knowledge has not been demonstrated before.

## Supplementary Material

SUPPLEMENTARY MATERIAL
