# Supplementary material for: Quantitative Assessment of Liver Function Using Gadoxetate-Enhanced Magnetic Resonance Imaging: Monitoring Transporter-Mediated Processes in Healthy Volunteers
Source: Invest Radiol. 2016 Dec 19;52(2):111–9. doi: 10.1097/RLI.0000000000000316 (PMC5228626; doi:10.1097/RLI.0000000000000316)
Supplement: SUPPLEMENTARY MATERIAL [file rli-52-111-s001.docx]

**Supplemental Digital Content**

The response of a compartment to an influx of contrast agent is assumed to be linear and stationary, i.e. proportional to the dose administered and independent of the time of arrival. No contrast agent is created or destroyed inside the compartment and therefore the rate of change of concentration is simply the sum of all outlets subtracted from the sum of all inlets^28,29,44,51^. This is described by equation 1:

$\frac{dC(t)}{dt}=\sum_{inlets} J_{i}\left( t \right)-\sum_{outlets} J_{o}\left( t \right)$ [1]

where *C(t)*, *J*_i_ and *J*_o_ denote the concentration time series of a compartment and the respective inlet and outlet fluxes. The measured liver concentration (*C*_l_) is equal to the weighted sum of the extracellular (*v*_ecs_) and intracellular compartments (*v*_i_) and is described by the following equations 2-4:

$v_{ecs}\frac{dc_{e}(t)}{dt}=\frac{F_{p}\left( f_{a}c_{a}\left( t \right)+f_{v}c_{v}\left( t \right) \right)}{1-Hct}-F_{p}c_{e}\left( t \right)-k_{i}c_{e}(t)$ [2]

$v_{i}\frac{dv_{i}(t)}{dt}=k_{i}c_{e}\left( t \right)-k_{ef}c_{i}(t)$ [3]

$C_{l}\left( t \right)= v_{ecs}c_{e}\left( t \right)+v_{i}c_{i}\left( t \right)$ [4]

where *c*_a_*(t)* and *c*_v_*(t)* are the concentrations of gadoxetate in the arterial and venous blood supply, and *Hct* is the haematocrit. Using convolution ($*$) the tissue concentrations can be expressed as

$C_{l}\left( t \right)=c_{p}(t)*I(t)$, $c_{p}\left( t \right)=(f_{a}c_{a}\left( t \right)+f_{v}c_{v}\left( t \right))/(1-Hct)$ [5]

$I\left( t \right)=F_{p}R(t)$, $R\left( t \right)=R_{e}\left( t \right)+R_{i}\left( t \right)$ [6]

$v_{ecs}c_{e}\left( t \right)=F_{p}c_{p}(t)*R_{e}(t)$ [7]

$v_{i}c_{i}\left( t \right)=F_{p}c_{p}(t)*R_{i}(t)$ [8]

where *R*_e_*(t)* and *R*_i_*(t)* are the residue functions for the extracellular and intracellular compartments, respectively. Using Laplace transform and initial condition *c*_e_*(t=0)*=0 the following equations for the extracellular compartment are obtained (equations 9-10):

$v_{ecs}sc_{e}\left( s \right)=F_{p}-F_{p}c_{e}\left( s \right)-k_{i}c_{e}\left( s \right)\leftrightarrow v_{ecs}c_{e}\left( s \right)=F_{p}\frac{1}{s+T_{e}^{-1}}$ [9]

$v_{ecs}c_{e}\left( s \right)=F_{p}R_{e}(s)$ [10]

where *T*_e_=*v*_ecs_/(*F*_p_+*k*_i_) is the extracellular compartment mean transit time. Comparing equations 9 and 10 and applying Inverse Laplace transform an expression for the extracellular residue function is obtained (equation 11):

$R_{e}\left( t \right)=e^{-t/T_{e}}$ [11]

Using a similar approach for the intracellular compartment, with initial condition *c*_i_*(t=0)*=0 and taking the Laplace transform of equations 3 and 8 the equations 12 and 13 are obtained:

$v_{i}sc_{i}\left( s \right)=k_{i}c_{e}\left( s \right)-k_{ef}c_{i}\left( s \right)\leftrightarrow v_{i}c_{i}\left( s \right)=\frac{E_{i}}{T_{e}}\cdot\frac{1}{s+T_{e}^{-1}}\cdot\frac{1}{s+T_{i}^{-1}}$ [12]

$v_{i}c_{i}\left( s \right)=F_{p}R_{i}(s)$ [13]

where *T*_i_=*v*_i_/*k*_ef_ and *E*_i_=*k*_i_/(*F*_p_+*k*_i_) are the intracellular mean residence time and intracellular uptake fraction (or extraction fraction), respectively. Comparing equation 12 and 13 and applying inverse Laplace transform the intracellular residue function is given by equation 14:

$R_{i}\left( t \right)=\frac{E_{i}}{1-T_{e}/T_{i}}\cdot(e^{-\frac{t}{T_{i}}}-e^{-\frac{t}{T_{e}}})$ [14]

Substituting equations 11 and 14 in 5 a solution for the liver tissue concentration is obtained (equation 15):

$C_{l}\left( t \right)=F_{p}\cdot\left\{ \frac{E_{i}}{1-\frac{T_{e}}{T_{i}}}\cdot e^{-\frac{t}{T_{i}}}+\left( 1-\frac{E_{i}}{1-\frac{T_{e}}{T_{i}}} \right)\cdot e^{-\frac{t}{T_{e}}} \right\}*c_{p}(t)$ [15]
