# Supplementary material for: Quantitative Assessment of Liver Function Using Gadoxetate-Enhanced Magnetic Resonance Imaging: Monitoring Transporter-Mediated Processes in Healthy Volunteers
Source: Invest Radiol. 2016 Dec 19;52(2):111–9. doi: 10.1097/RLI.0000000000000316 (PMC5228626; doi:10.1097/RLI.0000000000000316)
Supplement: SUPPLEMENTARY MATERIAL [file rli-52-111-s002.docx]

**Supplemental Digital Content 2**

Monitoring the *in vivo* distribution of gadoxetate

The volume slab acquired (Figure S2-1A) provided the ability to simultaneously monitor the distribution of the contrast agent in multiple organs including the liver, spleen and kidneys. The series of images acquired during the arterial phase (<5 min) demonstrate the rapid distribution of gadoxetate in major arteries such as the left renal artery, the superior mesenteric artery (SMA) and the abdominal aorta (Figure S2-1B). Images acquired during the hepatobiliary phase reveal additional anatomical regions that would otherwise be indistinguishable, such as the enhancement of the common bile duct (CBD), the gallbladder and the duodenum (Figure S2-1C). During the late hepatobiliary phase, when gadoxetate is mostly removed from the circulation, the inferior vena cava (IVC) and branches from the hepatic portal vein are delineated, due to their high contrast difference from the liver tissue (Figure S2-1D).

Examples of the resulting relative signal enhancement time series that can be extracted from the images are shown in Figure S2-2, for a single participant. The population average input functions (AIF and VIF) following bolus administration and distribution in the circulation are shown in Figure S2-3. Gadoxetate is removed from blood via a dual elimination pathway i.e. via the liver (Figure S2-2A) and kidneys (Figure S2-2B). Relative signal intensities in the spleen (Figure S2-2C) and kidneys, in conjunction with a rapid increase in signal intensity and fast wash-out, indicate that gadoxetate essentially remained extracellular in these organs. However, data showed accumulation of gadoxetate in the liver.

As demonstrated in previous *in vitro* studies gadoxetate is not expected to undergo passive hepatic uptake diffusion, but is more likely to exhibit active transport into hepatocytes due to its hydrophilic properties^1,2^. The fact that the contrast agent accumulated in the hepatocytes is not only evident from the prolonged enhancement throughout the scan duration, but also from the fact that the peak liver concentration was reached a substantial time after administration (>20 min), compared to extracellular contrast agents. Following the process of active uptake across the basolateral membrane, efflux transporters (e.g. MRP2) located on the apical membrane of hepatocytes are thought to actively transport gadoxetate into a network of channels that form the bile canaliculi^3^. These rigid ducts receive bile components secreted from liver cells, and via contractions drive their contents towards converging bile ducts that merge into the common bile duct^4^.

A representative signal from the CBD is shown in Figure S2-2D. Superior slices, closer to the liver, indicated that the proposed efflux mechanism starts only a few minutes after gadoxetate administration. This time lag between the administration of gadoxetate and its appearance in the common bile duct is the time required for the agent to reach the liver from the site of administration, be taken up into hepatocytes and subsequently effluxed into the canalicular space, and to be transportered to, and released from, the gallbladder (Figure S2-2E) into the CBD. The contrast agent is subsequently released from the CBD to the duodenum, whereby the agent is excreted (Figure S2-2E). The relative signal intensity time series obtained from the duodenum demonstrated an increase followed by a rapid decrease of the MRI signal, which was repeated four times during the latter 30 minutes of the scan. This profile is likely to be the result of peristalsis, which translocates the contents from the duodenum to a more distal region of the small intenstine. These findings highlight the possibility of using DCE-MRI techniques and the hepatobiliary contrast agent, gadoxetate, to monitor liver physiology and hepatobiliary function.


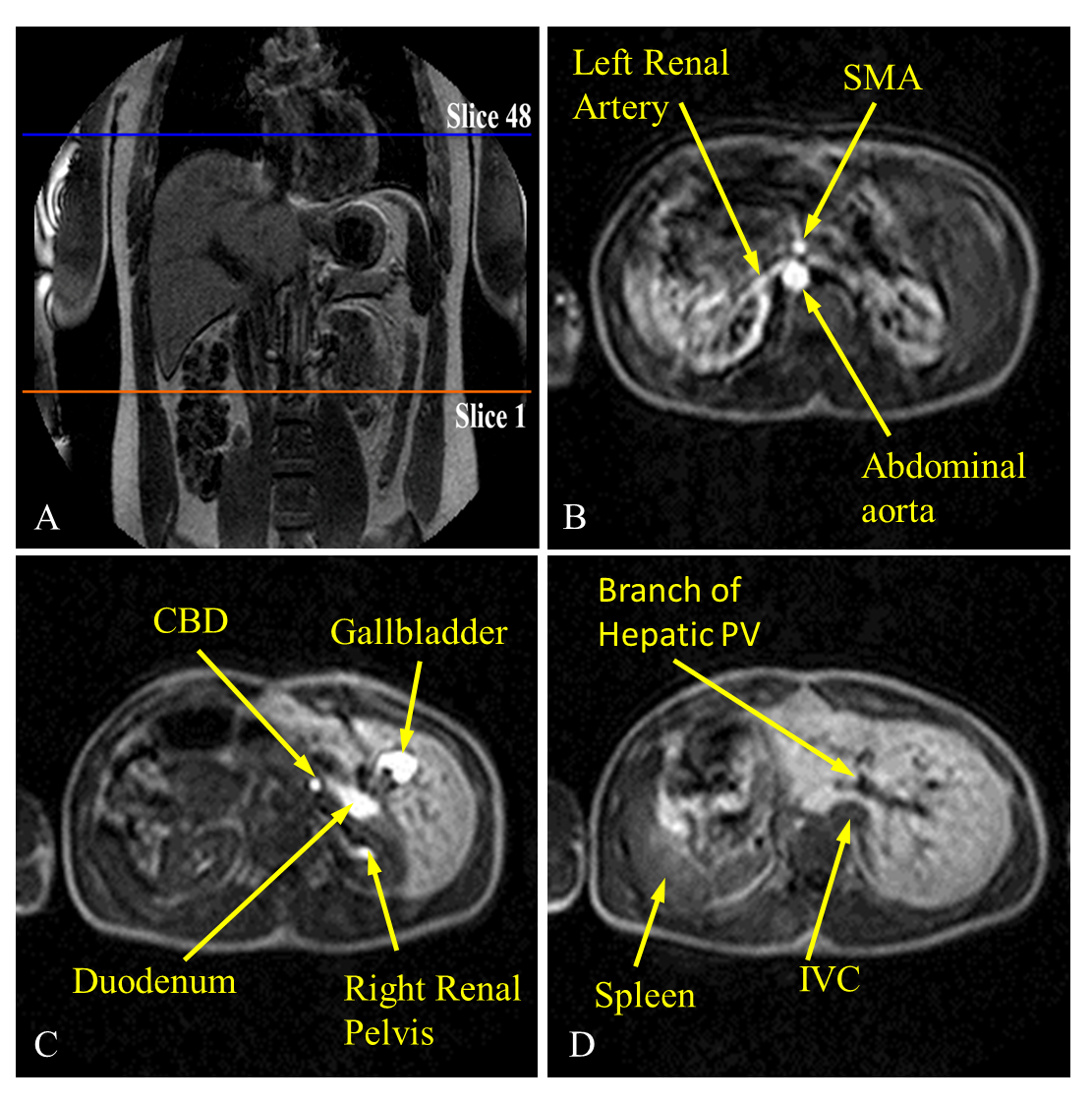


Figure S2-1. MR images of (A) a coronal slice acquired prior to contrast administration. Slice 1 and slice 48 correspond to the inferior and superior axial slices in the acquisition volume, respectively. Axial DCE-MR image of the 16^th^ slice of a healthy volunteer at the time of contrast administration, (B), demonstrating the enhancement of (i) abdominal aorta, (ii) left renal artery and (iii) the superior mesenteric artery (SMA). DCE-MR images acquired at the 25^th^ minute of the acquisition of slice 16 (C), demonstrating (i) gallbladder, (ii) common bile duct (CBD), (iii) right renal pelvis and (iv) duodenum, and slice 30 (D) demonstrating (i) inferior vena cava (IVC), (ii) a branch of the hepatic portal vein and (iii) spleen.


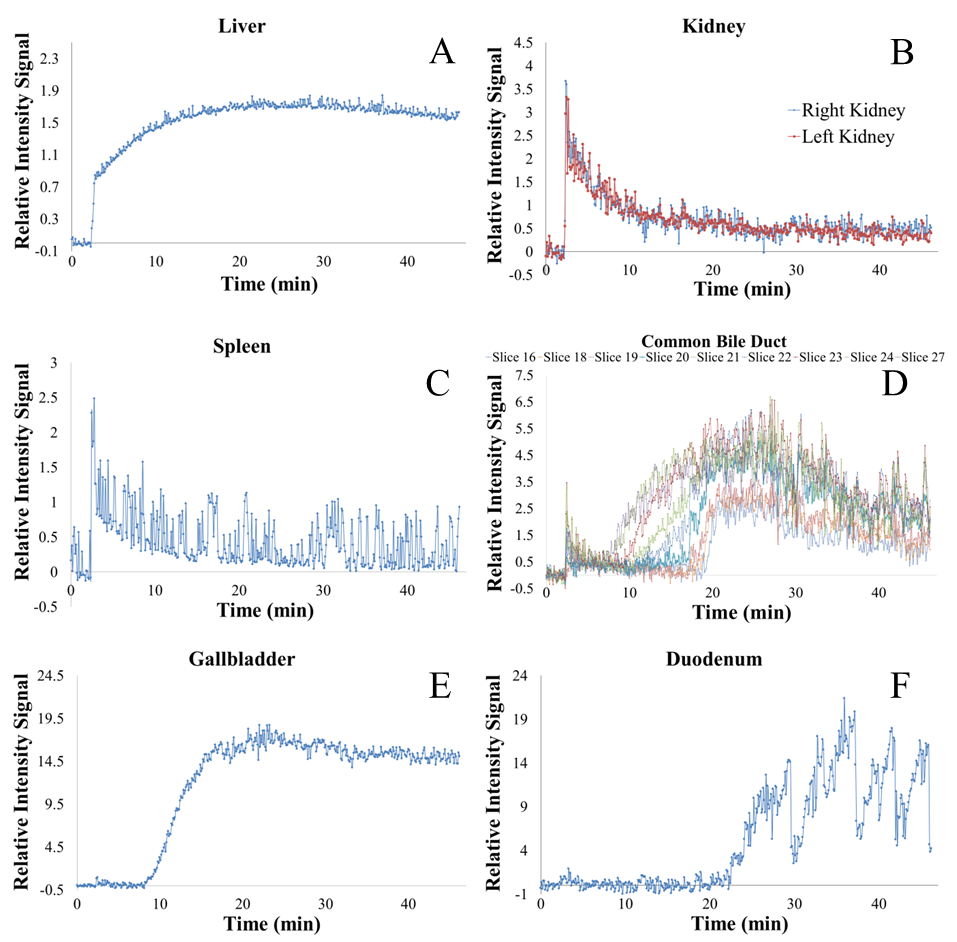


Figure S2-2. Relative signal intensity time series of (A) liver, (B) kidneys, (C) spleen, (D) common bile duct, (E) gallbladder and (F) duodenum from one participant.


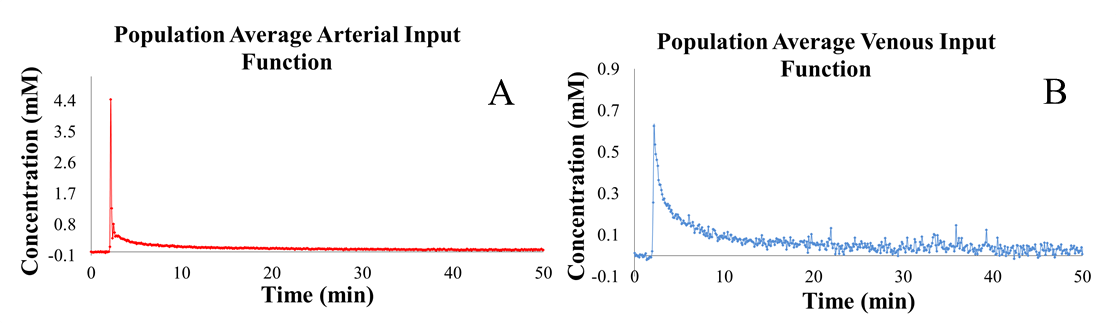


Figure S2-3. Population average (A) arterial input function generated from all individuals AIFs, (B) hepatic portal venous function generated from twelve individual VIFs.

**References**

1. Leonhardt M, Keiser M, Oswald S, et al. Hepatic uptake of the magnetic resonance imaging contrast agent Gd-EOB-DTPA: role of human organic anion transporters. *Drug Metab Dispos*. 2010;38(7):1024-1028.

2. Jia J, Keiser M, Nassif A, et al. A LC-MS/MS method to evaluate the hepatic uptake of the liver-specific magnetic resonance imaging contrast agent gadoxetate (Gd-EOB-DTPA) in vitro and in humans. *J Chromatogr B Analyt Technol Biomed Life Sci*. 2012;891-892:20-26.

3. Saito S, Obata A, Kashiwagi Y, et al. Dynamic contrast-enhanced MRI of the liver in Mrp2-deficient rats using the hepatobiliary contrast agent Gd-EOB-DTPA. *Invest Radiol*. 2013;48(7):548-553.

4. Watanabe N, Tsukada N, Smith CR, et al. Motility of bile canaliculi in the living animal: implications for bile flow. *J Cell Biol*. 1991;113(5):1069-1080.
